# Maleylpyruvic Acid-Inducible Gene Expression System and Its Application for the Development of Gentisic Acid Biosensors

**DOI:** 10.1021/acs.analchem.4c03906

**Published:** 2024-11-16

**Authors:** Ingrida Kutraite, Ernesta Augustiniene, Naglis Malys

**Affiliations:** 1 Bioprocess Research Centre, Faculty of Chemical Technology, Kaunas University of Technology, Radvilėnų Street 19, Kaunas LT-50254, Lithuania; 2 Department of Organic Chemistry, Faculty of Chemical Technology, Kaunas University of Technology, Radvilėnų Street 19, Kaunas LT-50254, Lithuania

## Abstract

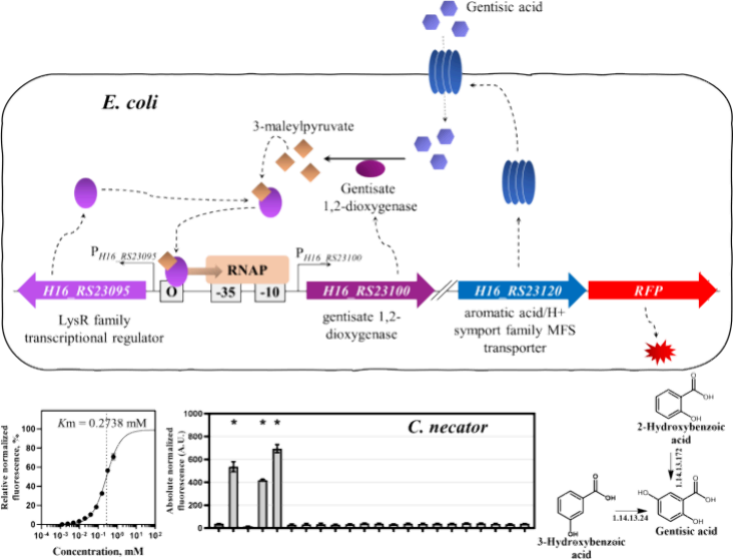

Gentisic acid is a secondary plant metabolite, known for its health benefits, not only widely used as a supplement but also implicated as a potential biomarker for cancer-associated metabolism alterations. To advance bioproduction and detection of this compound or its derivatives, cell-based approaches have become of interest in recent years. However, the lack of tools for high-throughput gentisic acid monitoring and compound-metabolizing organism screening limits the progress in this area. Here, we analyzed the gene cluster responsible for gentisic acid metabolism in *Cupriavidus necator* H16. The transcriptional regulator GtdR-based inducible gene expression system *Cn*GtdR/P_*gtdA*_ was elucidated, showing that it was activated when *C. necator* cells were subjected to gentisic acid. Subsequently, a 3-maleylpyruvic acid was identified as a primary inducer for this inducible system. Furthermore, genes *gtdA* and *gtdT*, encoding for gentisate 1,2-dioxygenase and MFS transporter, were shown to be essential for inducible system activation in the presence of gentisic acid with GtdA enabling conversion of this phenolic acid into the inducer. The *Cn*GtdRAT/P_*gtdA*_-based inducible system was employed to develop a whole-cell biosensor for the intracellular and extracellular detection of gentisic acid. The potential of the 3-maleylpyruvic acid-inducible system was demonstrated by its application in metabolic pathway research, detection of highly unstable 3-maleylpyruvic acid, and development of biosensors for the intracellular or extracellular determination of gentisic acid. In addition, the utility of the biosensor was emphasized by its application for detection of gentisic acid as a potential biomarker for cancer in urine samples.

## Introduction

Gentisic acid (2,5-dihydroxybenzoic acid) is one of the phenolic acids synthesized as secondary metabolites in plants. It is also found as a minor catabolite of aspirin in humans. This hydroxybenzoic acid exhibits many pharmacological activities and is recognized as an antioxidant, antimicrobial, anticarcinogen, analgesic, hepatoprotectant, neuroprotectant, nephroprotectant, and cardioprotectant.^1,2^ As a siderophore, it not is only essential for bacteria to sequester the limited iron from the environment but also can play a similar role in eukaryotic cells. Gentisic acid was shown to possess potential for cancer prevention,^3^ as it limits the availability of iron required for cancer cell proliferation.^2^ A higher retention of exogenous gentisic acid and its altered metabolic profile were observed in humans with cancer, making this phenolic acid a prospective biomarker for cancer detection.^4−9^

In the last few decades, gentisic acid has been broadly applied in material technology and synthesis. For example, in matrix-assisted laser desorption/ionization (MALDI), it is used as a component of matrix material contributing to the improved sensitivity and resolution.^10^ Gentisic acid and its esters find the application in cosmetics as skin-whitening substances for treatment of skin pigmentary disorders.^11^ It is used in the preparation of gentisic acid–gelatin conjugate, a polymer for the development of drug formulations.^12^ Gentisic acid is a precursor for synthesis of landomycin A, an angucycline antibiotic.^13^

The conventional methods of gentisic acid production involve carboxylation of hydroquinone^14^ and synthesis from 2-hydroxybenzoic acid via Elbs persulfate oxidation,^15^ or it can be obtained via plant extraction.^16^ However, the application of microbial production of gentisic acid and its pathway engineering are at the early stage of development.^17,18^

With increasing demand for biobased product synthesis, significant effort is dedicated to the microbial production of chemical compounds. To advance microbiology and bioproduction of gentisic acid or its derivatives and use gentisic acid as a potential biomarker for cancer detection, tools suitable for screening gentisate-producing strains and relevant pathway engineering are highly needed. Hence, this study focuses on the development of transcription-factor-based biosensors, which can be applied in the screens and used to monitor intracellular or extracellular levels of gentisic acid. Since no gentisic acid-sensing system has been characterized to date, a gene operon responsible for gentisic acid metabolism in *Cupriavidus necator* was studied. Subsequently, an inducible gene expression system responding to 3-maleylpyruvic acid (3-MPA) was identified and in combination with gentisate 1,2-dioxygenase used to develop a whole-cell biosensor for detection of gentisic acid. In addition, the applicability of the developed biosensor is demonstrated for intracellular and extracellular detection of gentisic acid, including urine samples.

## Results and Discussion

### Identification of *C. necator* H16-Inducible Gene Expression System Activated in the Presence of Gentisic Acid

Inducible gene expression systems are commonly composed of a gene encoding a transcriptional regulator (TR) and an inducible promoter, containing TR and RNA polymerase (RNAP) binding sites. The association or dissociation of TR with its binding site can either activate or repress RNAP-promoter complex formation and transcription. In addition, the binding of a ligand molecule to the TR can contribute to the activation or repression of an inducible system. Importantly, such inducible systems can respond to the ligand in a dose-dependent manner, providing opportunities for application in fine-tuning the gene expression or metabolite monitoring.^19^

**Figure 1:**
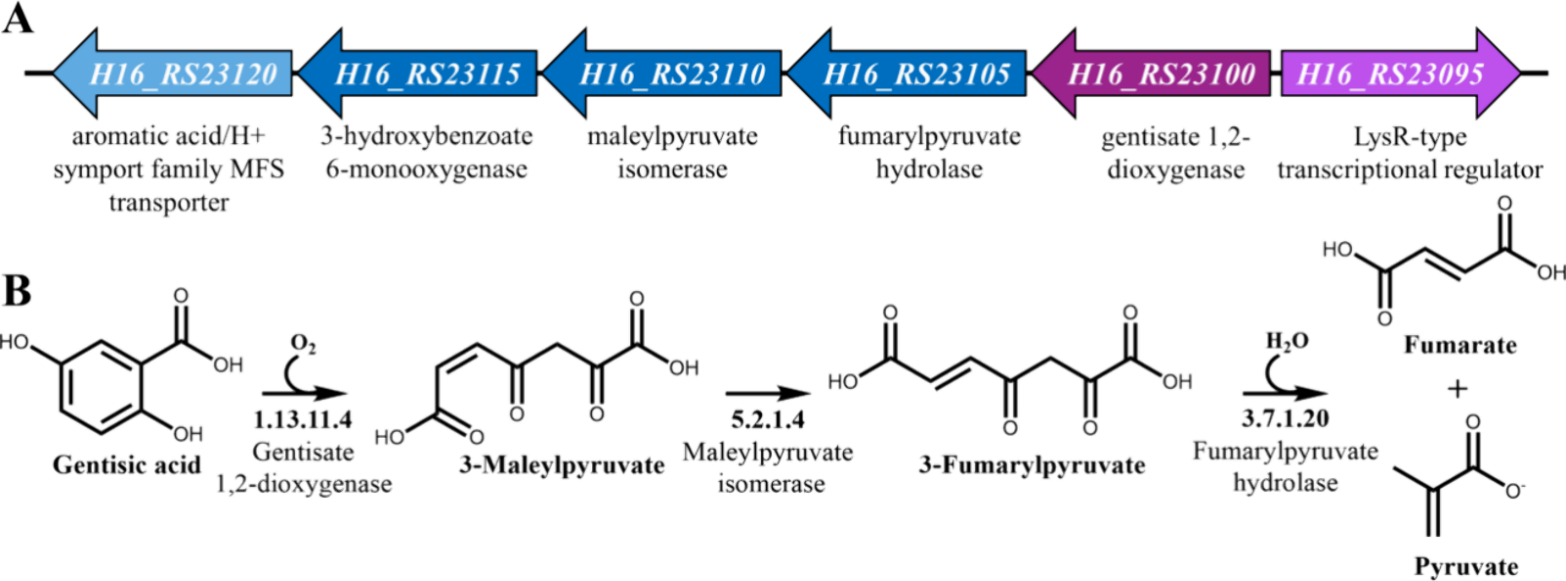
Gentisic acid catabolism in *C. necator* H16: (A) identified operon involved in the gentisic acid catabolism; (B) catabolic pathway of gentisic acid.

In a search for an inducible gene expression system that is activated in the presence of gentisic acid, we explored gene operons responsible for gentisic acid metabolism in bacteria. Gentisic acid catabolic pathways have been so far characterized in *Ralstonia* sp. U2,^20^*Klebsiella pneumoniae* M5a1,^21^*Pseudomonas* TA-2,^22^ and *Corynebacterium glutamicum* ATCC 13032.^23^ Due to its biodegradative capabilities, *C. necator* H16 has been identified as a source abundant in inducible systems.^24^ In this study, by applying a protein homology search, we identified that gentisate 1,2-dioxygenase encoding the *nagI* product from *Ralstonia* sp. U2 corresponds to 85% cover and 39.17% identity to the protein with locus tag *H16_RS23100* (*gtdA*) from *C. necator* H16, whereas *nagL* encoding maleylpyruvate isomerase corresponds to 99% cover and 51.17% identity to the one encoded by *H16_RS23110* (*maiA*). Figure 1A shows a corresponding *C. necator* H16 regulon composed of gentisate 1,2-dioxygenase (*gtdA*), fumarylpyruvate hydrolase (*H16_RS23105*), maleylpyruvate isomerase (*maiA*), 3-hydroxybenzoate 6-monooxygenase (*H16_RS23115*), aromatic acid/H+ symport family MFS transporter (*H16_RS23120*, hereafter referred to as *gtdT*), and LysR-type TR-encoding gene (*H16_RS23095*, hereafter referred to as *gtdR*), with the latter potentially responsible for regulation of operon expression.

As reviewed in ref (25), the 3-MPA conversion into pyruvic acid and maleic acid occurs through either glutathione-independent cleavage by maleylpyruvate hydrolase, or glutathione-dependent isomerization to 3-fumarylpyruvic acid (3-FPA) by maleylpyruvate isomerase and subsequent 3-FPA cleavage by fumarylpyruvate hydrolase, the pathway also reported in *Ralstonia* sp. U2.^20^

Figure 1B shows the proposed catabolic pathway for *C. necator* H16. Here, gentisic acid is converted to 3-MPA by gentisate 1,2-dioxygenase (EC 1.13.11.4), followed by isomerization to 3-FPA by glutathione-dependent maleylpyruvate isomerase (EC 5.2.1.4) and hydroxylation to fumaric acid and pyruvic acid by fumarylpyruvate hydrolase (EC 3.7.1.20). Notably, the gentisic acid catabolic pathway is a primary downstream route used by bacteria for aerobic catabolism of 3-hydroxybenzoic and 2-hydroxybenzoic acids.^26^*C. necator* H16 contains both 2-hydroxybenzoic and 3-hydroxybenzoic acid-specific enzymes,^27^ leading to the gentisic acid pathway.

**Figure 2:**
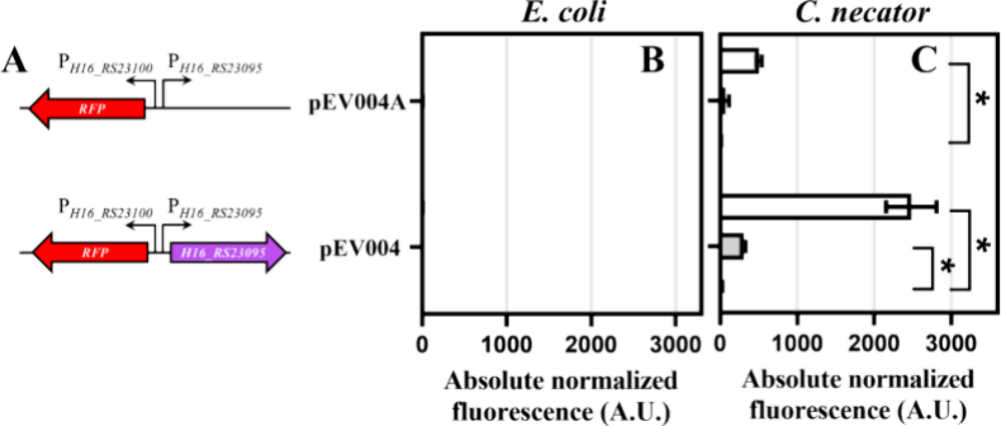
Development of the gentisic acid-inducible biosensors. (A) Genetic organization of the inducible system’s variants containing TR-encoding gene and promoter site, or promoter-only, originating from *C. necator* H16. Absolute normalized fluorescence measured in LB medium 6 h after exogenous addition of gentisic acid to the final concentration of 1.25 mM (white), 39 μM (light gray), and 0 mM (dark gray) in *E. coli* Top10 (B), or in *C. necator* H16 (C). Data represent mean values ± SD of three biological replicates, *p < 0.001 (unpaired *t* test).

To investigate whether the regulon of the identified catabolic pathway (Figure 1) contains a complete inducible gene expression system able to respond to gentisic acid, the constructs, carrying either an intergenic region *gtdR-gtdA* with a potential inducible promoter or inducible systems composed of TR gene *gtdR* and an intergenic region, were cloned into a RFP reporter vector. Resulting constructs with either *Cn*P_*gtdA*_ (pEV004A) or *Cn*GtdR/P_*gtdA*_ (pEV004), respectively, were tested in *Escherichia coli* and *C. necator* in the presence of gentisic acid (Figure 2). *C. necator* cells carrying either plasmid pEV004 or pEV004A displayed an increase in RFP synthesis of up to 2463- or 496-fold 6 h after supplementation with 1.25 mM of gentisic acid, and 298- or 67-fold with 39 μM of gentisic acid, respectively, whereas no response to this compound was observed in *E. coli* (Figure 2). These results indicated that the intergenic region *gtdR-gtdA* contains an inducible promoter (hereafter denoted as P_*gtdA*_) that is activated in *C. necator* when the gentisic acid is present in the growth medium. However, the mechanism of activation remained unclear, as no activation of this promoter was detected in *E. coli*.

### Elucidation of Mechanism and Genes Essential for the P_*gtdA*_ Promoter’s Activation in Non-Host Bacteria

**Figure 3:**
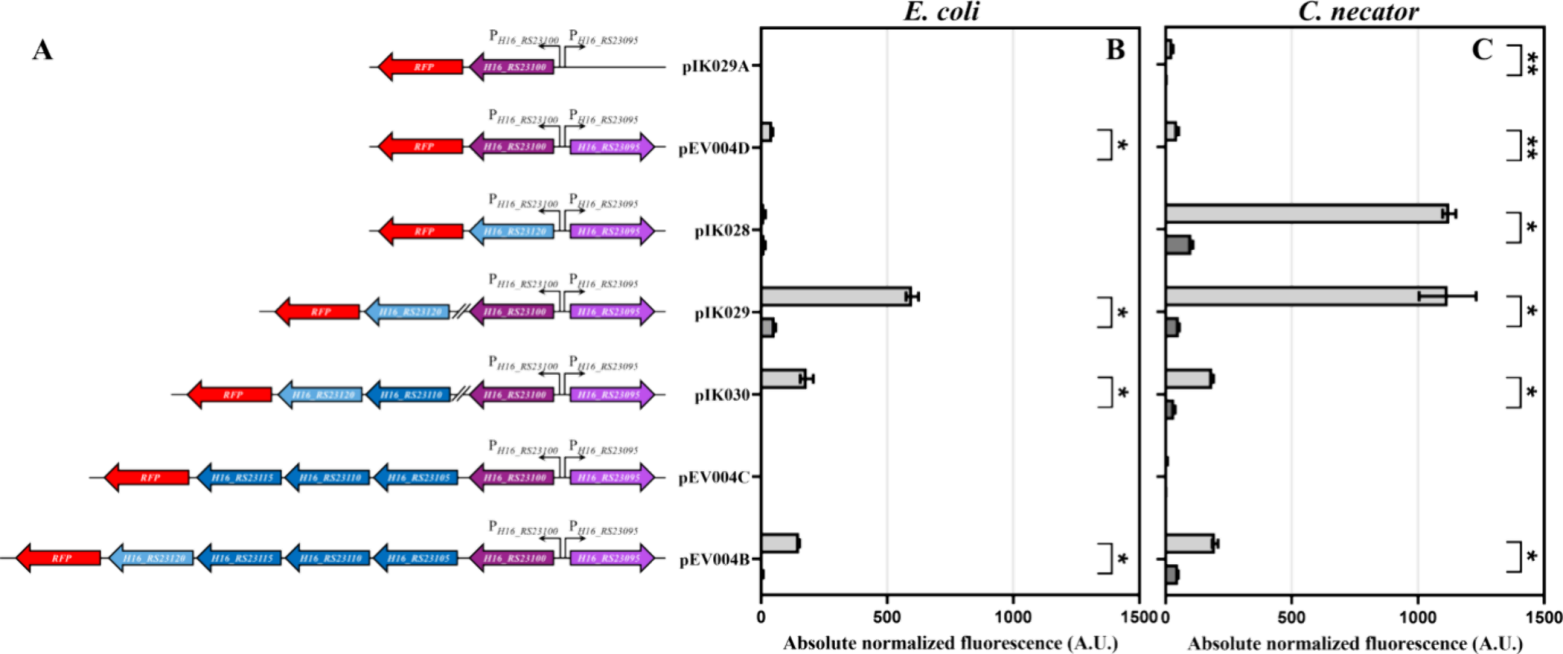
Development of the gentisic acid-inducible biosensors. (A) Genetic organization of the inducible system’s variants containing diverse sets of operon genes, originating from *C. necator* H16. Absolute normalized fluorescence measured in LB medium 6 h after exogenous addition of gentisic acid to the final concentration of 39 μM (light gray) and 0 μM (dark gray) in *E. coli* Top10 (B), or in *C. necator* H16 (C). Data represent mean values ± SD of three biological replicates, *p < 0.001, **p < 0.05 (unpaired *t* test).

To explain the absence of the P_*gtdA*_ promoter’s activity in *E. coli*, we hypothesized that either (a) additional regulatory factors are required for the promoter’s activation or/and specialized membrane transport proteins are required for uptake of gentisic acid into the cell, with both available only in *C. necator*, or (b) an intermediate of gentisic acid catabolism acts as an actual inducer. To further elucidate the mechanism and factors required for the activation of the P_*gtdA*_ promoter’s activation, gene subclusters of the gentisic acid catabolic operon, which included genes encoding aromatic acid/H+ symport family MFS transporter (*gtdT*), gentisate 1,2-dioxygenase (*gtdA*), maleylpyruvate isomerase (*maiA*), fumarylpyruvate hydrolase (*fmA*), or 3-hydroxybenzoate 6-monooxygenase (*hbA*), were assembled in combination with the reporter gene under transcriptional control of P_*gtdA*_ and *gtdR* (Figure 3A). *E. coli* strains carrying these constructs were grown in LB medium, and fluorescence output of the logarithmically growing cells was quantified 6 h after addition of gentisic acid. Remarkably, the inclusion of genes involved in gentisic acid catabolism enabled the activation of promoter P_*gtdA*_. Cells carrying constructs pEV004D, pIK029, pIK030, and pEV004B exhibited a statistically significant increase in absolute normalized fluorescence outputs after the addition of gentisic acid to the growth medium at the final concentration of 39 μM (Figure 3B). A closer analysis of the composition of these constructs revealed that in addition to TR gene *gtdR*, the *gtdA* gene was also present. No other tested genes were critical as transcriptional activation was observed in the case of construct pEV004D. These findings indicated that the gentisate 1,2-dioxygenase plays an indirect but essential role for the induction when cells are subjected to the inducer. Consequently, since the gentisate 1,2-dioxygenase can convert gentisic acid into 3-MPA (Figure 1B), we hypothesized that the latter, the catabolic intermediate of gentisic acid, is a “true” inducer of the *Cn*GtdR/P_*gtdA*_ system.

Additionally, cells carrying constructs pIK029, pIK030, and pEV004B with the *gtdT* gene encoding the aromatic acid/H+ symport family MFS transporter exhibited much higher activation of reporter gene expression than that of pEV004D (Figure 3B). Furthermore, constructs pIK030 and pEV004B containing the *maiA* gene encoding the maleylpyruvate isomerase showed a lower level of induction than that of pIK029, whereas pEV004C carrying *maiA* but not *gtdT* exhibited no detectable activation. Based on these results, we reasoned that GtdT enhances uptake of gentisic acid, whereas MaiA can rapidly reduce the intracellular concentration of the inducer by converting 3-MPA to 3-FP.

Notably, GtdT exhibits a significant similarity (38.61% identity and 92% coverage) to *E. coli* MhpT encoding for the 3-(3-hydroxyphenyl)propionate transporter. However, Xu reported that MhpT does not support the uptake of gentisate.^28^ On the other hand, a small fraction of aromatic acid, which will be present in the undissociated form under neutral or slightly acidic conditions of growth medium, can enter the cell by passive diffusion. This likely can explain the minor induction observed in the absence of the GtdT transporter in *E. coli* (pEV004D, Figure 3B).

*C. necator* strains harboring different versions of the inducible system–reporter construct showed a similar pattern of induction compared to that in *E. coli*, except pIK028 (Figure 3B,C). Although this construct does not possess *gtdA*, the genomic copy of this gene appears to be sufficient for the activation of the inducible system resulting in a 1021-fold increase in RFP synthesis that is similar to 1065-fold induction observed with pIK029. In addition, the critical role of gentisate dioxygenase GtdA in synthesis of the inducer was confirmed by knocking out *gtdA* in *C. necator* and plasmid-based complementation of the deletion (Supplementary Figure S1). These results further demonstrated that the *gtdA* gene is required for the activation of an inducible system, supporting the hypothesis that not the gentisic acid but its downstream catabolic intermediate 3-MPA acts as an inducer of *Cn*GtdR/P_*gtdA*_.

Similarly to the results obtained with *E. coli*, *gtdT* contributed to the enhancement of reporter gene expression in *C. necator* when it was introduced on the plasmid in addition to the chromosomal copy of this gene (Figure 3B,C). This suggested that the increase in the copy number of *gtdT* further contributed to the uptake of gentisic acid and/or the elevated intracellular concentration of the inducer (3-MPA), whereas, in the absence of an additional copy of *gtdT* on the plasmid (pIK029A, pEV004D, and pEV004C), the pace of gentisic acid catabolism likely exceeded the rate of gentisic acid uptake, and the transient concentration of the inducer (3-MPA) remained close or below the level required for the activation of reporter gene expression (Figure 3C). This was markedly pronounced when additional copies of gentisic acid catabolic pathway genes but not *gtdT* were introduced on the plasmid (pEV004C).

To date, a few catabolic pathway intermediate metabolites have been shown to play a role as a primary inducer of gene expression.^29−31^ Among these, CoA thioesters, kynurenine, and 4-oxalomesaconate have been shown to activate the catabolism of phenolic acids, L-tryptophan and gallic acid, respectively.^27,32,29^ The strategy to utilize the intermediate metabolite as a controller of dynamic pathway regulation has been applied in metabolic circuit design. For example, such approach has been implemented for the naringenin production using either an upstream biosensor that responds to *p*-coumaroyl-CoA based on the transcriptional repressor CouR from *Rhodopseudomonas palustris*(30) or a downstream sensor activated by kaempferol binding to the transcriptional repressor QdoR from *Bacillus subtilis*.^33,31^ Notably, intermediate metabolite-responsive biosensors provide an opportunity to test unstable compounds, which are often unavailable as analytical standards and are difficult to detect using common techniques.

Intriguingly, some studies have shown that primary and intermediate metabolites can act synergistically to induce gene expression. For example, benzoate and *cis*,*cis*-muconate can individually bind BenM, a LysR-type TR that controls aromatic compound degradation in *Acinetobacter baylyi* ADP1 and moderately activate the gene expression. However, only when both ligands act in conjunction and are bound to TR does the BenM achieve the conformation that enables the high-level transcriptional activation.^34,35^ A similar synergetic effect has been observed for binding of hydroxyphenylpropionate and phenylpropionate to MhpR TR from *E. coli* in the phenylpropionate catabolism activation.^36^

### Validation of 3-MPA as a Primary Inducer

**Figure 4:**
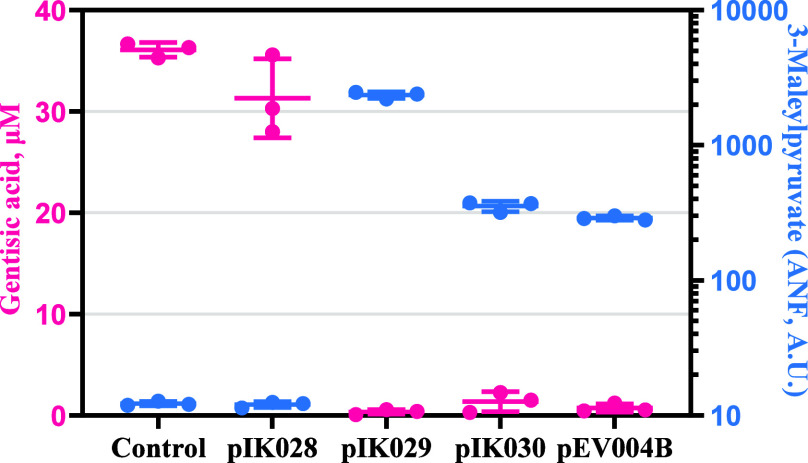
Comparison of gentisic acid consumption and fluorescence change 2 h after supplementation of 39 μM gentisic acid to *E. coli*-based biosensors. Absolute normalized fluorescence of *E. coli*-based biosensors (blue), amount of gentisic acid determined with HPLC in supernatant samples (pink), and data representing values ± SD of three biological replicates. Amount of gentisic acid determined with HPLC (left Y-axis), and intracellular amount of 3-MPA expressed as absolute normalized fluorescence (ANF, A.U.) (right Y-axis).

To further explore whether the intermediate 3-MPA of the gentisic acid catabolism is the primary inducer of *Cn*GtdR/P_*gtdA*_, logarithmically growing *E. coli* cells harboring constructs pIK028, pIK029, pIK030, and pIK004B were subjected to gentisic acid at the concentration 39 μM. Subsequently, supernatant and cell samples were collected for HPLC and single-time-point fluorescence analysis, respectively, at 0, 0.5, 2, 6, and 24 h after addition of gentisic acid (Supplementary Figure S2). Two hours after its addition, the gentisic acid was almost completely depleted in samples with *E. coli* strains carrying *gtdT* and *gtdA* (pIK029, pIK030, and pIK004B) (Figure 4), indicating that these genes enabled the uptake and biochemical transformation of this compound. Simultaneously, reporter gene expression was activated in these strains. Notably, the strains carrying a copy of *maiA* (pIK030 and pIK004B) exhibited nearly 10-fold lower gene expression activation than that of strain with pIK029. Moreover, more rapid consumption of gentisic acid was measured in the sample with construct pEV004B containing the fumarylacetoacetate hydrolase gene (Supplementary Figure S2C) compared to that of pIK030. Altogether, these data showed that gentisate dioxygenase GtdA enabled the transformation of gentisic acid into the inducer, whereas maleylpyruvate isomerase MaiA reduced the latter’s transient concentration by converting it into a downstream product. Therefore, based on the above observations and known catalytic activities of gentisate dioxygenase and maleylpyruvate isomerase (Figure 1B), it can be concluded that the inducer is 3-MPA.

Next, the conversion of gentisic acid to 3-MPA by GtdA was investigated in the cell extracts prepared from *E. coli* carrying constructs pIK028 (*gtdA*^*–*^) and pIK029 (*gtdA*^*+*^). Gentisic acid was added to the extracts, and absorbance at 320 nm for gentisic acid and 330 nm for 3-MPA was measured as described previously.^20,37^ The absorbance shift from 320 to 330 nm was observed in the extract prepared from *gtdA*^*+*^ cells, whereas the control strain *gtdA*^*–*^ showed no such change (Supplementary Figure S3).

**Figure 5:**
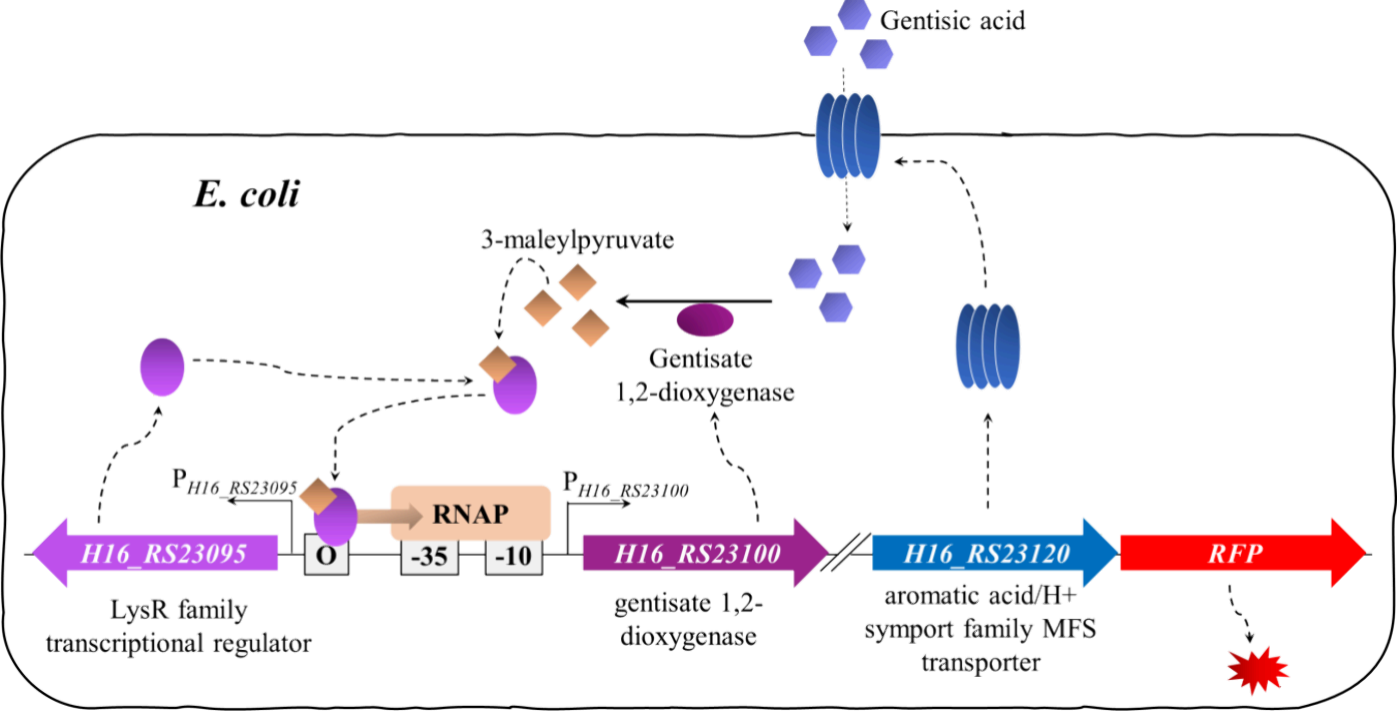
A visual representation of the *E. coli*-based *Cn*GtdRAT/P_*gtdA*_ biosensor mechanism of action. The flow of gentisic acid through the generated MFS transporter induces the expression of gentisate 1,2-dioxygenase, which converts gentisic acid to 3-MPA, subsequently generating a complex with TR GtdR, which induces the action of RNA polymerase (RNAP) by connecting to the operator site and therefore provides positive feedback by enhancing the expression of gentisate 1,2-dioxygenase and the MFS transporter, along with RFP.

Altogether, the obtained results showed that the intermediate metabolite 3-MPA of the gentisic acid catabolism is the primary inducer of the *Cn*GtdR/P_*gtdA*_-inducible gene expression system. Moreover, in combination with aromatic acid/H+ symport family MFS transporter GtdT and gentisate 1,2-dioxygenase GtdA, this system can be used as an *E. coli*-based gentisic acid biosensor. The mechanism of action for such a biosensor, hereafter referred to as *Cn*GtdRAT/P_*gtdA*_-based *E. coli* biosensor, is shown in Figure 5.

### Parameterization of the Gentisic Acid Biosensor

**Figure 6:**
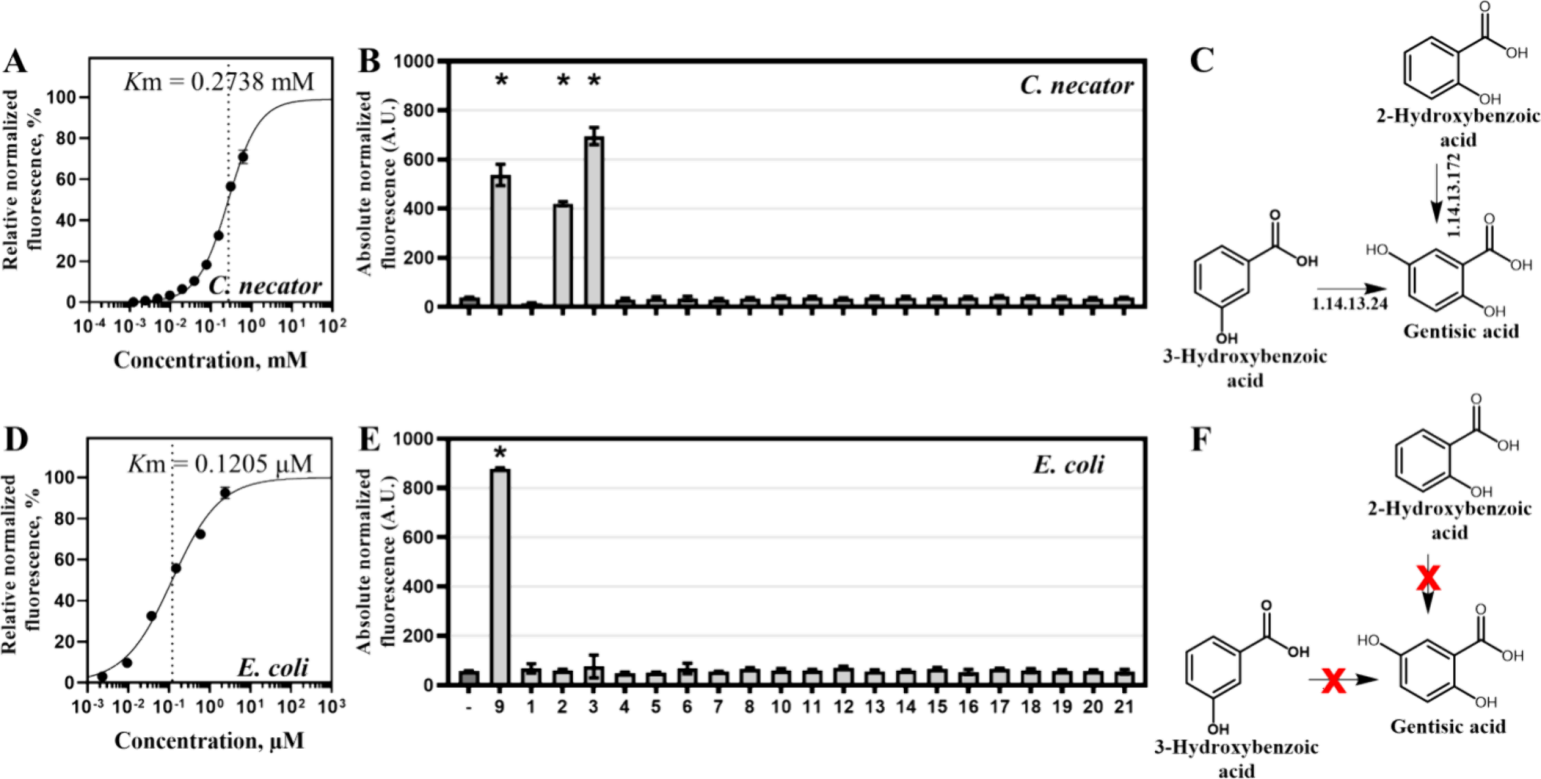
Parameterization of the *Cn*GtdRAT/P_*gtdA*_-based biosensors. Dose–response curves of *C. necator* (A) and *E. coli* (D) biosensors 6 h after the addition of different concentrations of gentisic acid, ranging from 0 to 0.625 mM and from 0 to 2.4 μM, respectively. The dose–response curves were fitted using the Hill function, as described in Materials and Methods. *K*_m_ is indicated by a dotted line. Data represent mean values ± SD of three biological replicates. *C. necator* (B) or *E. coli* (E) sensor specificity was evaluated by determining the absolute normalized fluorescence in LB medium 6 h after supplementation with various phenolic acids to the final concentration of 9.75 μM. Compounds that were tested for cross-reactivity with biosensors were the following: 4-hydroxybenzoic acid (1), 2-hydroxybenzoic acid (2), 3-hydroxybenzoic acid (3), vanillic acid (4), isovanillic acid (5), gallic acid (6), protocatechuic acid (7), syringic acid (8), gentisic acid (9), α-resorcylic acid (10), β-resorcylic acid (11), γ-resorcylic acid (12), orsellinic acid (13), 6-methylsalicylic acid (14), *o*-coumaric acid (15), *m*-coumaric acid (16), *p*-coumaric acid (17), ferulic acid (18), sinapic acid (19), caffeic acid (20), and chlorogenic acid (21). Data represent mean values ± SD of three biological replicates, *p < 0.001 (unpaired *t* test). The metabolic network between 2-hydroxybenzoic acid (2), 3-hydroxybenzoic acid (3), and gentisic acid (9) in *C. necator* (C) and *E. coli* (F).

In addition to the characterization of the molecular mechanism, the dynamics and ligand specificity of the *Cn*GtdRAT/P_*gtdA*_-based biosensor was investigated. First, the dose–response relationship between the concentration of extracellularly added gentisic acid and fluorescence output was examined in *C. necator* and *E. coli* (Figure 6A,D). The *C. necator*-based biosensor was tested in the range of 0 to 0.625 mM, whereas the *E. coli* version of the sensor was subjected to the gentisic acid concentration of up to 39 μM only. At higher concentrations of gentisic acid, the growth-inhibitory effect was observed for both types of whole-cell biosensors (Supplementary Figure S4). Since *E. coli* does not possess the pathway for 3-MPA metabolism, the accumulation of this compound likely contributed to the reduced tolerance of gentisic acid compared to *C. necator*. The dose–response curve of the *C. necator*-based biosensor indicated that the gene expression can be tuned in the range of approximately 4.8 μM to 0.625 mM, whereas the *E. coli* sensor exhibited a differential response for gentisic acid concentrations of 9.52 nM to 2.4 μM (Figure 6A,D). Notably, the *K*_m_ values differed significantly between *C. necator* and *E. coli* sensors (0.2738 and 0.1205 μM). Similarly, the limit of detection (LOD), representing the lowest concentration of inducer that can activate the system, was also divergent, 0.152 μM and 9.52 nM, respectively. Such differentiation can be explained by the absence of the 3-MPA metabolic pathway in *E. coli* and its presence in *C. necator*, where this compound can be readily transformed reducing the transient concentration of the inducer and the sensor’s sensitivity.

Next, other phenolic acids were investigated for cross-reactivity with *Cn*GtdRAT/P_*gtdA*_-based *E. coli* and *C. necator* biosensors. Single-time-point fluorescence measurements were performed 6 h after compounds were supplemented at a final concentration of 9.75 μM. The statistically significant (*p* < 0.001) activation of reporter gene expression by 2-hydroxybenzoic, 3-hydroxybenzoic, and gentisic acids was observed using the *C. necator*-based biosensor (Figure 6B,C). In this case, we identified enzymes that catalyze the conversion of 2-hydroxybenzoic acid and 3-hydroxybenzoic acid into gentisic acid, which is subsequently transformed into the primary inducer of the gene expression system, 3-MPA. The salicylate 5-hydroxylase (EC 1.14.13.172) encoded by *nagIHG* (*H16_RS08115-H16_RS08125*) was proposed to act on 2-hydroxybenzoic acid, whereas 3-hydroxybenzoate 6-monooxygenase (EC 1.14.13.24, gene locus tag *H16_RS23115*) hydroxylated the 3-hydroxybenzoic acid to gentisic acid (Figure 6C). Notably, the delay in the sensor’s response was evident with 2-hydroxybenzoic acid and 3-hydroxybenzoic acid (Supplementary Figure S5), indicating the extended time required for transformation of these compounds to gentisic acid followed by conversion to 3-MPA. The *E. coli* sensor exhibited response to gentisic acid only (Figure 6E,F). In this instance, a higher 821-fold induction was observed compared to that of *C. necator* (498-fold), likely reflecting the accumulation of 3-MPA in *E. coli*.

The information collated in Supplementary Table S1 highlights outstanding characteristics of whole-cell biosensors in comparison to technologies reported for the detection of gentisic acid. The *Cn*GtdRAT/P_*gtdA*_*-*based *E. coli* biosensor showed a very high sensitivity to this compound with the LOD in the lower range of nM concentration, which outperformed most of other techniques and was only matched by HPLC-MS/MS-based application.^38^

### Application of Biosensor for Gentisic Acid Detection in Urine

**Figure 7:**
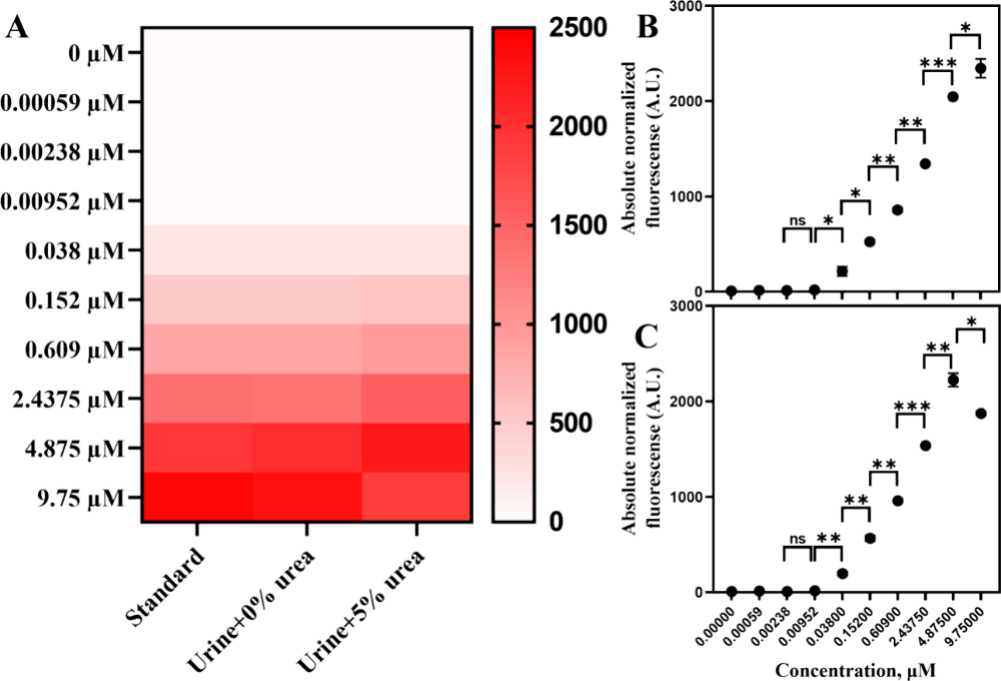
Application of the *Cn*GtdRAT/P_*gtdA*_-based *E. coli* biosensor for the detection of gentisic acid in the urine samples. (A) Heat map illustrates activation of reporter gene expression 2 h after addition of diverse concentrations of gentisic acid, ranging from 0 to 9.75 μM, dissolved in the water (standard) or in the synthetic urine with 0% urea or synthetic urine with 5% urea. Single-time-point induction values displayed in absolute normalized fluorescence 2 h after addition of various concentrations of gentisic acid dissolved in either synthetic urine with 0% urea (B) or synthetic urine with 5% urea (C). Measurements were performed in LB medium, ± SD of two technical replicates, *p < 0.05; **p < 0.01; ***p < 0.001 (unpaired *t* test).

Some cancers are difficult to detect due to absence of symptoms.^39^ Gentisic acid is retained in cancer patients due to organism metabolic changes.^8^ Renal cell carcinoma (RCC) is known as one of the deadliest urogenital cancers, and it is among the 10 most common cancers worldwide.^40^ Altered levels of gentisic acid were reported in the urine of RCC patients compared to the healthy group.^9^ To demonstrate the utility of the developed whole-cell biosensor, the determination of gentisic acid in synthetic and artificial urine samples was chosen as a proof-of-concept approach for detection of this compound. Both types of urine samples supplemented with various concentrations of gentisic acid were subjected to the analysis using the *Cn*GtdRAT/P_*gtdA*_-based *E. coli* biosensor. Since urea is known to interfere with usual detection screens, such as HPLC/MS, by resulting in a large peak that overlaps with the metabolite of interest,^41^ the test samples were challenged with up to 5% of urea. Results showed that the biosensor developed in this study was able to achieve an outstanding LOD of 9.52 nM for detection of gentisic acid in both urea-free and with 5% urea-supplemented synthetic urine samples with urea having a minor effect on the sensor’s performance (Figure 7). No statistically significant differences were observed in biosensor behavior when artificial urine was used in the assay compared to synthetic urine.

Compared to other technologies that were recently used for gentisic acid detection in urine,^42,43^ only the advanced HPLC-MS/MS technique was able to match and exceed the sensitivity of the biosensor by achieving an LOD of 0.324 nM.^44^ This demonstrated that the *Cn*GtdRAT/P_*gtdA*_-based *E. coli* biosensor can be used as a highly sensitive technology and potentially help overcome the limits of diagnosis. Considering that several publications have identified gentisic acid as a potential biomarker in RCC, the developed biosensor provides alternative technology for measuring this compound in urine.^4,5,7,8,44^ It should be noted that the quantity of this phenolic acid in urine is highly dependent on the patient diet or drug uptake.^4,7^ In particular, uptake of aspirin contributes to drastically elevated levels of gentisic acid.^4^ Importantly, the concentrations of this phenolic acid differ significantly between healthy human and RCC patient samples. A higher level of gentisic acid in RCC patients compared to a healthy group, 1457 ng/mL (9.75 μM) of gentisic acid in RCC patients, and 645 ng/mL (4.18 μM) in healthy patients were observed by Chen et al.^44^ In this study, the authors also noted that a higher number of patients and controls are required to validate gentisic acid as a diagnostic biomarker. Despite this, the statistical analysis does provide a solid support for high accuracy of this compound’s measurements in urine. Notably, the average concentration of gentisic acid in healthy adult urine (4.18 μM) reported previously^44^ indicates that the developed biosensor with a linear range of sensitivity from 9.52 nM to 2.4 μM (Figure 7B,C) provides a level of sensitivity sufficient for detection of this compound in more than 100 times diluted urine samples. This significantly reduces any possible interference or inhibitory effect on the biosensor by urea or proteins that are present in the samples of both healthy and cancer patients.

## Conclusions

The utility of inducible gene expression systems has been demonstrated in developing whole-cell biosensors and genetic circuit control. Gentisic acid has been widely acknowledged as a nutraceutical with several health benefits, and it has been recently proposed as a potential biomarker for cancer detection. To advance research into the detection and application of this compound *in situ*, we report here the characterization of an inducible gene expression system that is activated by 3-MPA, the downstream intermediate of gentisic acid metabolism. Furthermore, by cointegrating gentisate 1,2-dioxygenase and MFS transporter genes *gtdA* and *gtdT* with the 3-MPA-inducible *Cn*GtdR/P_*gtdA*_ system, the gentisic acid whole-cell *C. necator* and *E. coli* biosensors were developed and thoroughly characterized. The *C. necator*-based sensor was shown to be useful not only for sensing directly 3-MPA and indirectly gentisic acid but also for detection of 2-hydroxybenzoic and 3-hydroxybenzoic acids, whereas the *E. coli* version demonstrated high specificity and sensitivity to gentisic acid only. We proved the applicability of such sensor by using it for determination of gentisic acid in the urine samples as a potential biomarker in noninvasive cancer detection. This is the first report of the whole-cell biosensor suitable for the detection of gentisic acid.

## Materials and Methods

### Chemicals, Bacterial Strains, and Media

All chemicals used as inducers in this study are provided in Supplementary Table S2. All strains used in this study are provided in Supplementary Table S3. Of which, *E. coli* Top10 was used for cloning and plasmid propagation; *E. coli* Top10, *C. necator* H16, and *C. necator*Δ*gtdA* were used as hosts for fluorescence assays; and genomic DNA of *C. necator* H16 was used as a template to amplify DNA fragments containing genetic elements of the gentisic acid-inducible system. *E. coli* S17-1 was used for conjugative plasmid transfer. Cells were cultivated in Luria–Bertani (LB) medium, and antibiotics were added to the medium at the following concentrations: 25 or 50 μg/mL chloramphenicol for *E. coli* Top10 and *C. necator* H16, respectively, and 12.5 μg/mL tetracycline for *E. coli* S17-1. Solid medium was prepared by supplementation with 15 g/L agar. Synthetic urine was prepared as described for synthetic urine concentrate (Cat. No. 8362, RICCA Chemical) by obtaining the following composition: 0.1% MgSO_4_·7H_2_O, 0.06% CaCl_2_·2H_2_O, 0.727% NaCl, and respective amounts of urea (0% or 5%). Artificial urine was prepared according to ref (45) and included the following: 0.5% Na_2_HPO_4_, 0.75% NaCl, 0.45% KCl, 0.2% creatinine, 0.005% albumin, and respective amounts of urea (0% or 5%). Both types of urine samples were adjusted to pH 7.0 and filtered through a 0.22 μm filter.

### Cloning Ant Transformation

Plasmid DNA was purified using the GeneJET Plasmid Miniprep Kit (Thermo Fisher Scientific). Microbial genomic DNA was extracted by employing a GenElute Bacterial Genomic DNA Extraction Kit (Sigma-Aldrich). To derive the gel-purified linearized DNA, the Zymoclean Gel DNA Recovery Kit (Zymo Research) was used, and the NEBuilder HiFi DNA Kit was applied to assemble plasmids and was purchased from New England Biolabs. Phusion High-Fidelity DNA polymerase, DreamTaq DNA polymerase, and all restriction enzymes, AatII, NdeI, SacI, and SbfI, were purchased from Thermo Fisher Scientific. All reactions were set up according to the manufacturer’s protocol. Chemically competent *E. coli* cells were prepared and transformed using a heat-shock method as described in ref (46). *C. necator* cells were made electrocompetent and transformed using the electroporation method as described in ref (47).

### Plasmid Construction and Conjugative Gene Knockout

All plasmids were constructed using the NEBuilder HiFi DNA assembly master mix according to the manufacturer’s protocol (New England Biolabs) by cloning PCR-amplified DNA fragments into the AatII- and NdeI-digested pBRC1 vector,^48^ which was built as described for pEH006 in ref (49). All plasmids constructed and used in this study are listed in Table 1. Primers required for plasmid assembly are provided in Supplementary Table S4, and the information for construction of plasmids is provided in Supplementary Methods. The conjugation procedure was performed as described by ref (50).

**Table 1:** Plasmids Used in This Study

**plasmid**	**characteristic**	**reference**
**pBRC1**	*Cm*^*r*^; modular vector for the evaluation of inducible systems; P_*araC*_-*araC*-T*rrnB1* and P_*araBAD*_-T7sl-EcRBS-*rfp*-T_*dbl*_	(49)
**pLO3**	Tet^*r*^; modular vector for the assembly of conjugative vector	(51)
**pEV004**	*Cm*^*r*^; P_*gtdR*_-*gtdR*-T_*rrnB1*_ and P_*ccl*_-*rfp*-T_*dbl*_ from *C. necator* H16 genomic DNA	(27)
**pEV004A**	*Cm*^*r*^; P_*gtdR*_-T_*rrnB1*_ and P_*gtdA*_-*rfp*-T_*dbl*_ from *C. necator* H16 genomic DNA	(27)
**pIK028**	*Cm*^*r*^; P_*gtdR*_-*gtdR*-T_*rrnB1*_, P_*gtdA*_-*gtdA*-*fmT*-*maiA*-*hbA*-*gtdT*-*rfp*-T_*dbl*_ from *C. necator* H16 genomic DNA	this study
**pIK029**	*Cm*^*r*^; P_*gtdR*_-*gtdR*-T_*rrnB1*_, P_*gtdA*_-*gtdA*-*fmT*-*maiA*-*hbA*-*rfp*-T_*dbl*_ from C. necator H16 genomic DNA	This study
**pIK030**	*Cm*^*r*^; P_*gtdR*_-*gtdR*-T_*rrnB1*_, P_*gtdA*_-*gtdA*-*rfp*-T_*dbl*_ from C. necator H16 genomic DNA	this study
**pEV004B**	*Cm*^*r*^; P_*gtdR*_-*gtdR*-T_*rrnB1*_, P_*gtdA*_-*gtdT*-*rfp*-T_*dbl*_ from C. necator H16 genomic DNA	this study
**pEV004C**	*Cm*^*r*^; P_*gtdR*_-*gtdR*-T_*rrnB1*_, P_*gtdA*_-*gtdA*-*gtdT*-*rfp*-T_*dbl*_ from C. necator H16 genomic DNA	this study
**pEV004D**	*Cm*^*r*^; P_*gtdR*_-*gtdR*-T_*rrnB1*_, P_*gtdA*_-*gtdA*-*maiA*-*gtdT*-*rfp*-T_*dbl*_ from C. necator H16 genomic DNA	this study
**pIK029A**	*Cm*^*r*^; P_*gtdA*_-*gtdA*-*rfp*-T_*dbl*_ from C. necator H16 genomic DNA	this study
**pIK040**	Tet^*r*^; conjugative vector to perform *gtdA*-knockout from C. necator H16 genomic DNA	this study

### Absorbance and Fluorescence Analyses

For evaluation of absolute normalized fluorescence, plasmid-transformed bacterial cells were grown overnight in 2 mL of LB medium containing appropriate antibiotics with orbital shaking at 200 rpm and 30 °C. Afterward, the precultures were diluted 50 times into a fresh LB medium with respective antibiotics and were grown with 200 rpm orbital shaking at 30 °C in 50 mL conical tubes. The 142.5 μL of cells with an absorbance of 0.1–0.2 was transferred to a 96-well microtiter plate (flat and clear bottom, black, VWR International) and supplemented with 7.5 μL of inducer to achieve a final concentration as indicated.

RFP fluorescence was determined by using an Infinite M200 PRO (Tecan) microplate reader. The RFP fluorescence was measured using 585 nm as excitation wavelength and 620 nm as emission wavelength, with 9 and 20 nm band widths, respectively. Measurements were taken at intervals of 18 min for a time course of 20 h. The gain factor was set to 120%. Parallelly, the absorbance was measured using a wavelength of 600 nm with a 9 nm bandwidth. The obtained values were normalized by calculating absolute normalized fluorescence (ANF), as described previously^19^^1^:
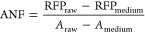
1where RFP_raw_ and *A*_raw_ are the absolute fluorescence and absorbance values of culture; RFP_medium_ and *A*_medium_ are the absolute fluorescence and absorbance values of the medium.

Subsequently, biosensors were parametrized by evaluating the relative normalized fluorescence and applying a nonlinear least-squares fitting to the Hill function. The values of ANF were calculated and plotted as a function of effector concentration using the software GraphPad Prism 9, using formula 2:
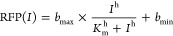
2where RFP(*I*) is the ANF value at given effector concentration *I*; *b*_max_ and *b*_min_ are the maximum and minimum levels of reporter output in ANF units, respectively; *h* is the Hill coefficient; and *K*_m_ is the inducer concentration, corresponding to the half-maximal reporter’s output.

The dynamic range indicating the fold of induction was calculated either by subtracting the ANF of the uninduced sample value from the ANF of the induced sample value, or by dividing *b*_max_ by *b*_min_, when the latter parameters were estimated for the dose–response analysis.

### HPLC Analysis

HPLC analysis of consumed gentisic acid in biosensor culture supernatants was performed by using an UltiMate 3000 HPLC system equipped with a photodiode array (UV–Vis) detector (Thermo Fisher Scientific). Chromatographic separation was achieved with a Phenomenex Luna 5 μm C18 100 Å (150 × 4.60 mm) column equipped with a Phenomenex Security Guard Cartridge (part number KJ0-4282), thermostated at 25 °C. Mobile phase A was aqueous 0.1% formic acid (v/v), and mobile phase B was HPLC-grade acetonitrile. The elution gradients used were as follows: from 0 until 15 min from 10 to 50% B, from 15 to 17.5 min raised at 70% B, and from 17.5 to 20 min decreased to 10% B and then kept constant for 2 min. A constant flow rate of 1 mL/min was maintained throughout the analysis with the detection wavelength set at 260 nm. The samples were filtered using a 0.22 μm syringe filter, 10 μL of sample was injected, and the elute was detected at a wavelength of 320 nm. All chromatograms were recorded and analyzed using Chromeleon 7 software (Thermo Fisher Scientific).

### Gentisic Acid Transformation to 3-MPA

The overnight cell cultures were washed twice with fresh LB medium, diluted to 0.05–0.1 OD_600_, and grown to an OD of 0.5. Then, gentisic acid was added to a final concentration of 9.75 μM to induce the expression of gentisate 1,2-dioxygenase, and the cell were grown for an additional 2 h. Subsequently, the extraction using BugBuster mixture (Sigma-Aldrich) was performed in the presence of 0.05 mM NH_4_Fe(SO_4_)_2_·12H_2_O to provide Fe^2+^ ions for maintaining the stability of dioxygenase.^37^ The spectrophotometric measurement of 10 times diluted extract was performed in Na–K phosphate buffer (pH 7.4) supplemented with 0.078 mM of gentisic acid and 0.05 mM of NH_4_Fe(SO_4_)_2_·12H_2_O. Prior to the addition of extract, the reaction buffer was preincubated in a cuvette at 30 °C. The GtdA activity of gentisic acid was assayed by measuring the absorbance shift from 320 to 330 nm.

### Statistical Analysis

All data provided in this study are mean ± SD, *n* = 2 or *n* = 3. The statistical analysis was carried out by using GraphPad Prism 9.0, using an unpaired two-tailed *t* test to compare the means; the *p*-values of 0.05, 0.01, or 0.001 were considered significant.

## Supporting Information Available

Methods, method comparison for determination of gentisic acid, materials, experimental details, and results including data on induction in *C. necator*ΔgtdA knockout strain, consumption of gentisic acid by *E. coli*-based biosensor variants, activity of enzyme extract from *E. coli* harboring gentisate dioxygenase gene, and sensitivity of *E. coli* and *C. necator* CnGtdRAT/P_*gtdA*_-based biosensors to gentisic acid (PDF)
