# Correction to “Maleylpyruvic Acid-Inducible Gene Expression System and Its Application for the Development of Gentisic Acid Biosensor”

**DOI:** 10.1021/acs.analchem.6c03841

**Published:** 2026-06-30

**Authors:** Ingrida Kutraite, Ernesta Augustiniene, Naglis Malys

The currently published sentence on page 18732 “Results showed that the biosensor developed in this study was able to achieve an outstanding LOD of 9.52 nM for detection of gentisic acid in both urea-free and with 5% urea supplemented synthetic urine samples with the urea having a minor effect on sensor’s performance (Figure 7)” should be corrected to “Results showed that the biosensor developed in this study was able to achieve an outstanding LOD of 38.0 nM for detection of gentisic acid in both urea-free and with 5% urea supplemented synthetic urine samples with the urea having a minor effect on sensor’s performance (Figure 7)” with “38.0 nM” instead of “9.52 nM” used in this sentence.

Also, the currently published sentence on the page 18733 “Notably, the average concentration of gentisic acid in healthy adult urine (4.18 μM) reported previously^44^ indicates that the developed biosensor with linear range of sensitivity from 9.52 nM to 2.4 μM (Figure 7B,C) provides a level of sensitivity sufficient for detection of this compound in more than 100 times diluted urine samples” should be corrected to “Notably, the average concentration of gentisic acid in healthy adult urine (4.18 μM) reported previously^44^ indicates that the developed biosensor with linear range of sensitivity from 38.0 nM to 4.9 μM (Figure 7B,C) provides a level of sensitivity sufficient for detection of this compound in more than 100 times diluted urine samples” with “38.0 nM to 4.9 μM” instead of “9.52 nM to 2.4 μM” used in this sentence.

