# Supplementary material for: Maleylpyruvic Acid-Inducible Gene Expression System and Its Application for the Development of Gentisic Acid Biosensors
Source: Anal Chem. 2024 Nov 16;96(47):18727–35. doi: 10.1021/acs.analchem.4c03906 (PMC11603403; doi:10.1021/acs.analchem.4c03906)
Supplement: Supplementary file 1 — ac4c03906_si_001.pdf [file ac4c03906_si_001.pdf]

Supporting information

Maleylpyruvic acid-inducible gene expression system and its application for the development of gentisic acid biosensor

Ingrida Kutraite[1], Ernesta Augustiniene[1], and Naglis Malys[1,2]*

[1]Bioprocess Research Centre, Faculty of Chemical Technology, Kaunas University of Technology, Radvilėnų street 19, LT-50254 Kaunas, Lithuania

[2]Department of Organic Chemistry, Faculty of Chemical Technology, Kaunas University of Technology, Radvilėnų street 19, LT-50254 Kaunas, Lithuania

*Author to whom correspondence should be addressed; E-mail: n.malys@gmail.com

Supplementary Methods

Construction of plasmids

pIK004 was constructed by using oligonucleotide primers EV006 and EV007 to amplify 3-MP-inducible system from *C. necator* H16 genomic DNA, and cloned into pBRC1 vector by AatII and NdeI restriction sites.

pEV004A was constructed by using oligonucleotide primers EV006A and EV007 to amplify 3-MP-inducible promoter from *C. necator* H16 genomic DNA, and cloned into pBRC1 vector by AatII and NdeI restriction sites.

pIK028 was constructed by using oligonucleotide primers EV007B and IK062, IK061 and EV006 to amplify 3-MP-inducible system from *C. necator* H16 genomic DNA, and cloned into pBRC1 vector by AatII and NdeI restriction sites.

pIK029 was constructed by using oligonucleotide primers EV007B and IK063, IK064 and EV006 to amplify 3-MP-inducible system from *C. necator* H16 genomic DNA, and cloned into pBRC1 vector by AatII and NdeI restriction sites.

pIK030 was constructed by using oligonucleotide primers EV007B and IK057, IK058 and IK059, IK060 and EV006 to amplify 3-MP-inducible system from *C. necator* H16 genomic DNA, and cloned into pBRC1 vector by AatII and NdeI restriction sites.

pEV004B was constructed by using oligonucleotide primers EV006 and EV007F, EV007E and EV007B, to amplify 3-MP-inducible system from *C. necator* H16 genomic DNA, and cloned into pBRC1 vector by AatII and NdeI restriction sites.

pEV004C was constructed by using oligonucleotide primers EV006 and EV007C to amplify 3-MP-inducible system from *C. necator* H16 genomic DNA, and cloned into pBRC1 vector by AatII and NdeI restriction sites.

pEV004D was constructed by using oligonucleotide primers EV006 and EV007D to amplify 3-MP-inducible system from *C. necator* H16 genomic DNA, and cloned into pBRC1 vector by AatII and NdeI restriction sites.

pIK029A was constructed by using oligonucleotide primers EV006A and EV007D to amplify 3-MP-inducible system from *C. necator* H16 genomic DNA, and cloned into pBRC1 vector by AatII and NdeI restriction sites.

pIK040 was constructed by using oligonucleotide primers IK098 and IK099, IK100 and IK101 to amplify homology arms from *C. necator* H16 genomic DNA, and cloned into pLO3 vector by SacI and SbfI restriction sites, to obtain genomic knockout of gentisate 1,2-dioxygenase-encoding gene from genomic DNA of *C. necator*.

Supplementary Tables

Supplementary Table S1. Methods for determination of gentisic acid

Method	LOD[a]	LOQ[b]	Range	Note	Reference
HPLC-FRAP	2.5954 µM	8.425 µM	2.58-58.32 µM	Complicated sample preparation	1
Sensor based on an amino-montmorillonite-modified inkjet-printed graphene electrode	0.33 µM	n.d.	1-21 µM	Limited selectivity	2
LC/MS/MS	0.2 µM	2.79 µM	Up to 6.48 µM	Complicated sample preparation. Expensive method	3
RP-HPLC-DAD	0.1946 µM	0.7137 µM	0.7137-324.4 µM	Complicated sample preparation	4
HPLC/MS/MS	0.0033 µM	0.011 µM	0.065-32.44 µM	Quantification of gentisate is complicated. Demand for sample preparation. Expensive method	5
*Cn*GtdRAT/P_{gtdA} -based *E. coli* biosensor	0.00952 µM	n.d.	0.00952-2.4 µM	Specific method, high-throughput, real-time detection. Directly detects 3-MP and indirectly gentisate	This study
*Cn*GtdRAT/P_{gtdA} -based *C. necator* biosensor	0.15 µM	n.d.	0.0048-0.625 mM	High-throughput, real-time detection. Directly detects 3-MP and indirectly gentisate, 3-hydroxybenzoic, 2-hydroxybenzoic acids	

[a]limit of detection, representing a minimum analyte concentration that could be accurately identified
[b]limit of quantification, representing a minimum analyte concentration that could be accurately quantified

Supplementary Table S2. Chemicals used in this study.

Chemical	Supplier	Catalog number
Gallic acid hydrate	Fluorochem	242843
Sodium salicylate	Sigma-Aldrich	71945
Sodium 4-hydroxybenzoate	Fluorochem	047887
3-Hydroxybenzoic acid	Sigma-Aldrich	H20008
Vanillic acid	Sigma-Aldrich	94770
Isovanillic acid	Alfa Aesar	A13709
Protocatechuic acid	Alfa Aesar	B24016
Syringic acid	Alfa Aesar	A11725
Gentisic acid	Sigma-Aldrich	149357
α-Resorcylic acid	Sigma-Aldrich	D110000
β-Resorcylic acid	Sigma-Aldrich	D109401
γ-Resorcylic acid	Sigma-Aldrich	D109606
o-Orsellinic acid hydrate	Acros Organics	A0411384
6-Methylsalicylic acid	Acros Organics	341500
o-Coumaric acid	Sigma-Aldrich	H22809
m-Coumaric acid	Sigma-Aldrich	H23007
p-Coumaric acid	Sigma-Aldrich	C9008
Ferulic acid	Sigma-Aldrich	128708
Sinapic acid	Alfa Aesar	A15676
Chlorogenic acid	TCI	C0181

Supplementary Table S3. Strains used in this study.

Strains and plasmids	Characteristic	Supplier
Escherichia coli Top10	F^- *mcrA* Δ(*mrr-hsdRMS-mcrBC*) Φ80*lacZ*ΔM15 Δ*lacX*74 *recA*1 *araD*139 Δ(*araleu*)7697 *galU* *galK rpsL* (StrR) *endA1 nupG*	Thermo Fisher Scientific
Cupriavidus necator H16	Wild type strain DSM 428	DSMZ
E. coli S17-1 λpir	*thi pro hsdR*- *hsdM* + *recA* RP4-2-Tc:: Mu-Km::Tn7 λpir Tp^R Sm^R	[6]
*C. necator*Δ*gtdA*	Δ*gtdA* (Δ*H16_RS23100*)	This study

Supplementary Table S4. Oligonucleotide primers used in this study. Restriction sites are underlined.

Primer name	Primer sequence (5' → 3')
EV006	GGGCCTTTCGTTTTATGACGTCCAGAGTCTCCCGATCTGGAC
EV006A	GGGCCTTTCGTTTTATGACGTCGAAGCTACCTATATAAAAAAAC
EV007	CGTCTTCGCTACTCGCCATATGGTCTCCGTGACTTTTGCAGG
EV007B	CGTCTTCGCTACTCGCCATATGTATATCTCCTTCTTAAAAGATCTTTTGAATCAGA CTGCCAGGCGCTC
EV007C	CGTCTTCGCTACTCGCCATATGGGTCTCCTCCTTGATGCCAG
EV007D	CGTCTTCGCTACTCGCCATATGGGCAGGTCGGATCAGGG
EV007E	CCCGACTACACCGCCTGACG
EV007F	TCAGGCGGTGTAGTCGGG
IK057	AGGAGACAAGCATGAGCAGTTGGCTGTAAGCCGCTGTCGCAGC
IK058	ACTGCTCATGCTTGTCTCCT
IK059	GGAAACCTCGAACCATGTCCTACCGCGTGGTCTGAACCTCTG
IK060	GTAGGACATGGTTCGAGGTTTCC
IK061	GTCTCCGTGACTTTTGCAGG
IK062	CCTGCAAAAGTCACGGAGACATGCCTGCCAGCCAAC
IK063	ATGCCTGCCAGCCAAC
IK064	GTTGGCTGGCAGGCATGTCGCGAGACGCTCAGGT
IK098	TCTTCACCTAGATCCTTTTAATTCGAGCTCGACACCGAGCCGAAC
IK099	GGTGTGTTCGGACATGCT
IK100	AGCATGTCCGAACACACCCGCACCTGAGCGTCTCGC
IK101	AATTAGCTTGCATGCCTGTCGCGGACGGGTTGAT

Supplementary Figures

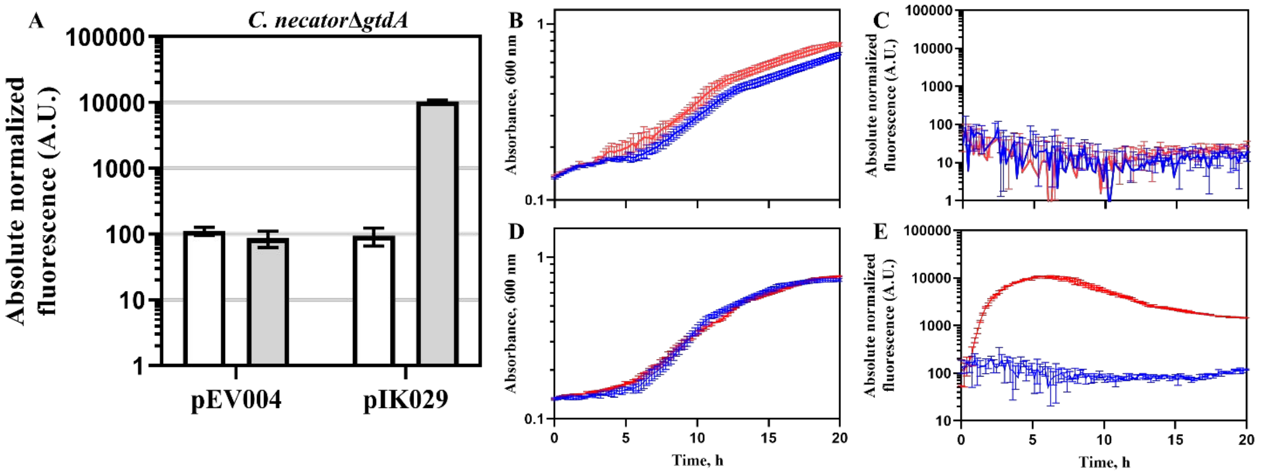

Supplementary Figure S1. Induction results of *C. necator*Δ*gtdA* knockout strain in LB medium. (A) Results of absolute normalized fluorescence using *C. necator* H16Δ*gtdA* carrying *Cn*GtdR/P$_{gtdA}$ (pEV004) or *Cn*GtdRAT/P$_{gtdA}$ (pIK029) inducible systems, 6 hours after exogenous addition of 1.25 mM (grey) or 0 mM gentisic acid (white). Absorbance at 600 nm of *C. necator*Δ*gtdA* carrying *Cn*GtdR/P$_{gtdA}$ (B) and *Cn*GtdRAT/P$_{gtdA}$ (D) inducible systems. Absolute normalized fluorescence of *C. necator*Δ*gtdA* carrying *Cn*GtdR/P$_{gtdA}$ (C) and *Cn*GtdRAT/P$_{gtdA}$ (E), using 585 nm as excitation wavelength and 620 nm as emission wavelength. Uninduced (blue), 1.25 mM gentisic acid (red). Data represent mean values of three biological replicates ± SD.

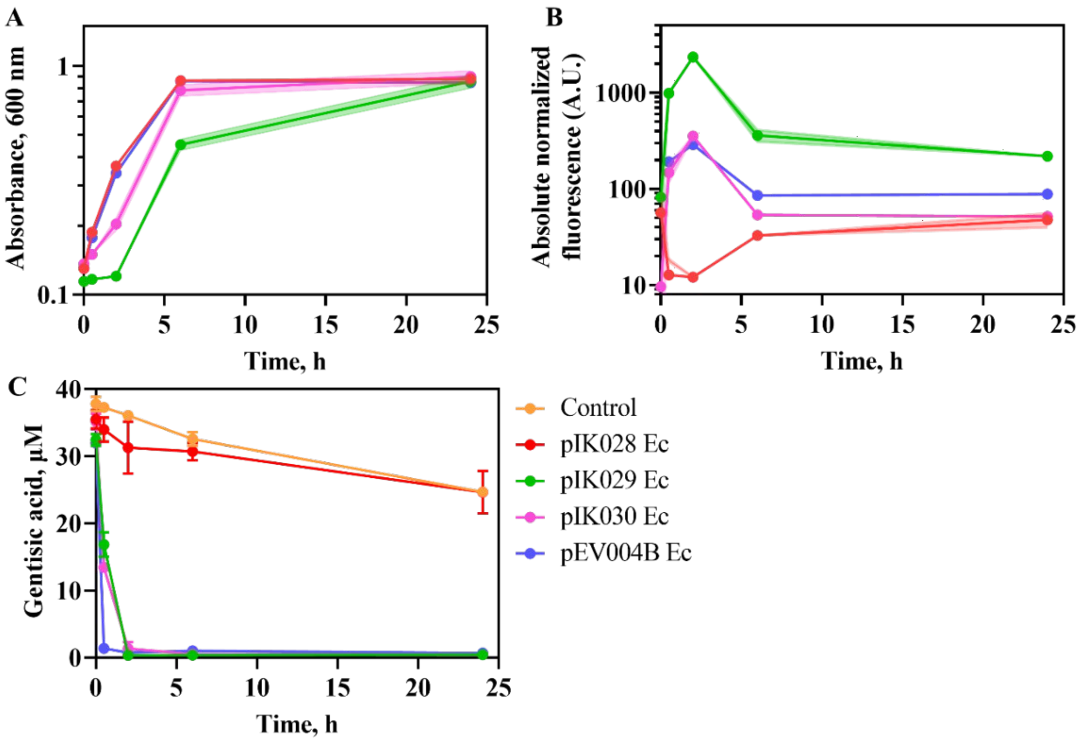

Supplementary Figure S2. Consumption of gentisic acid by *E. coli*-based gentisic acid-inducible biosensor variants. Absorbance at 600 nm (A) and absolute normalized fluorescence (B) of biosensor variants. (C) Amount of gentisic acid determined with HPLC in supernatant samples collected and measured at respective time points of 0, 0.5, 2, 6, and 24 hours. Measurements were taken using 39 μM of gentisic acid added at 0 hours, data represent mean values ± SD of three biological replicates.

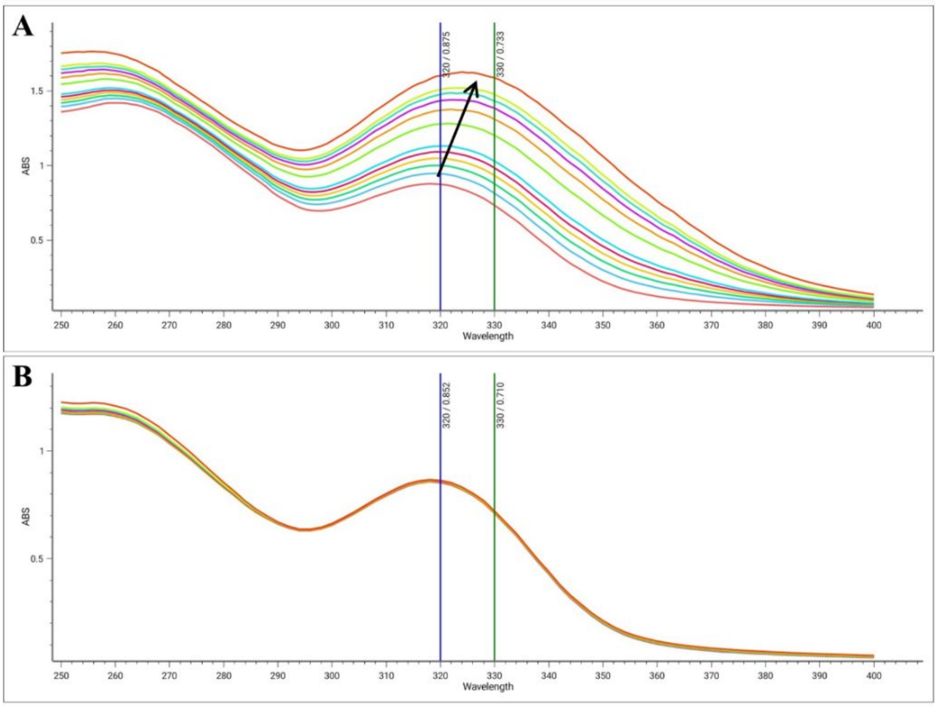

Supplementary Figure S3. The activity of enzyme extract from *E. coli* carrying either construct pIK029 containing MFS transporter (*gtdT*) and gentisate dioxygenase (*gtdA*) genes (A) or pIK028, as negative control, (B) was measured in a final volume of 1.0 mL containing Na-K phosphate buffer pH 7.4, 0.078 mM of gentisic acid, 0.05 mM $NH_4Fe(SO_4)_2x12H_2O$, and 100 µL of fresh extraction supernatant.

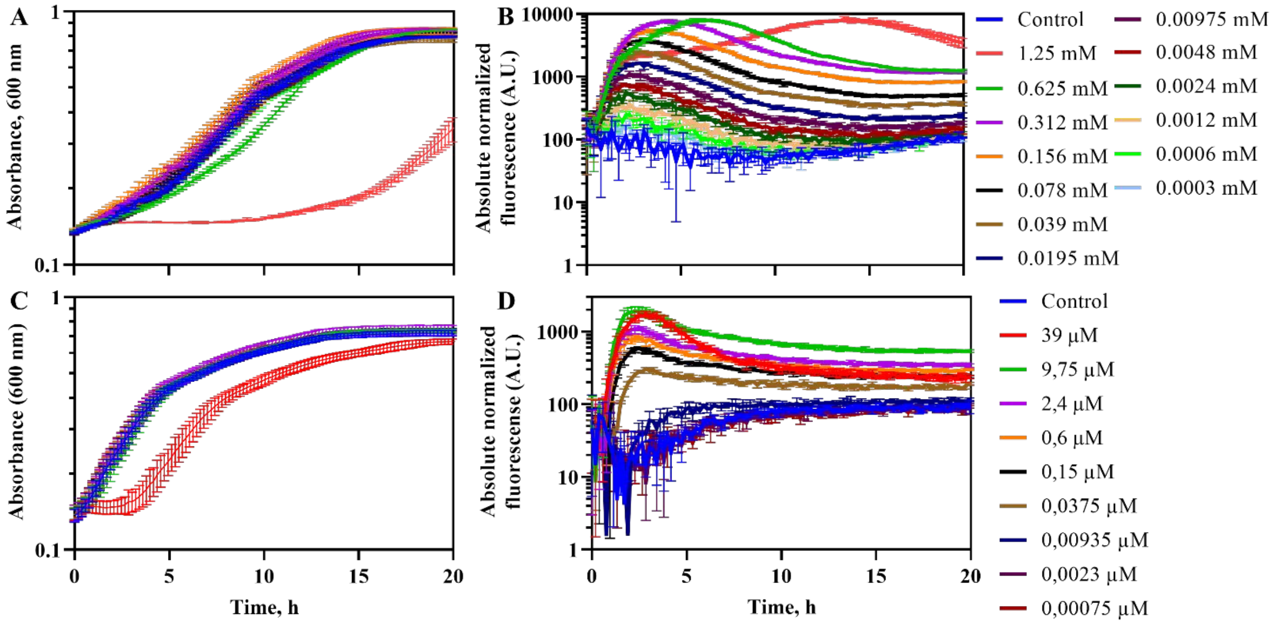

Supplementary Figure S4. The sensitivity of *Cn*GtdRAT/P$_{gtdA}$-based biosensors to gentisic acid of concentrations ranging from 0 to 39 µM and from 0 to 0.625 mM for *E. coli* and *C. necator*, respectively, measured in LB medium. Absorbance at 600 nm using *C. necator* (A) and *E. coli* (C) as hosts. Absolute normalized fluorescence using *C. necator* (B) and *E. coli* (D), using 585 nm as excitation wavelength and 620 nm as emission wavelength. Data represent mean values of three biological replicates ± SD.

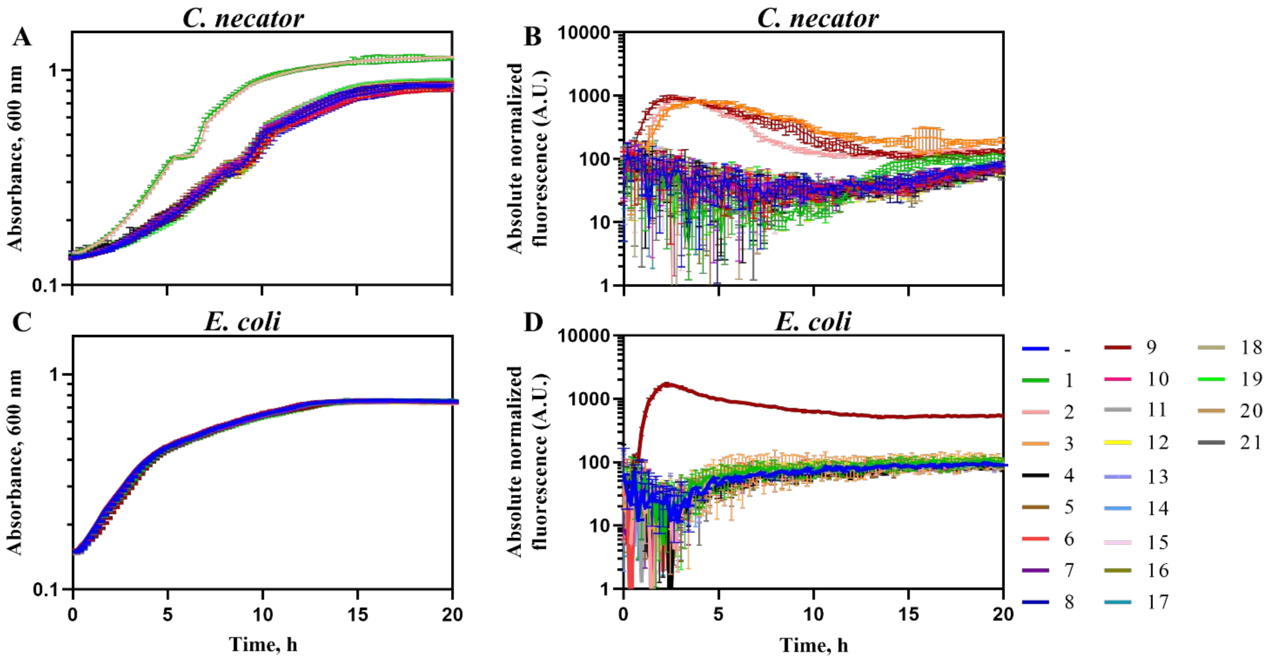

Supplementary Figure S5. The analysis of specificity of *C. necator*- and *E. coli*-based biosensors based on *Cn*GtdRAT/P_{gtdA} inducible system towards different phenolic acids. Absorbance of *C. necator* (A) and *E. coli* (C) and absolute normalized fluorescence of *C. necator* (B) and *E. coli* (D) biosensors were measured every 10 min for 20 hours after following phenolic acids were added to the cell culture at the final concentration of 9.75 µM: 4-hydroxybenzoic acid (1), 2-hydroxybenzoic acid (2), 3-hydroxybenzoic acid (3), vanillic acid (4), isovanillic acid (5), gallic acid (6), protocatechuic acid (7), syringic acid (8), gentisic acid (9), *α*-resorcylic acid (10), *β*-resorcylic acid (11), *γ*-resorcylic acid (12), orsellinic acid (13), 6-methylsalicylic acid (14), *o*-coumaric acid (15), *m*-coumaric acid (16), *p*-coumaric acid (17), ferulic acid (18), sinapic acid (19), caffeic acid (20), chlorogenic acid (21). Data represent mean values of three biological replicates ± SD.

References

1. Sinan, K. I. *et al.* HPLC-FRAP Methodology and Biological Activities of Different Stem Bark Extracts of *Cajanus cajan* (L.) Millsp. *JPBA* **2021**, *192* (113678).

2. Dongmo, L. M. *et al.* A New Sensor Based on an Amino-Montmorillonite-Modified Inkjet-Printed Graphene Electrode for the Voltammetric Determination of Gentisic Acid. *Microchim. Acta* **2021**, *188* (36).

3. Croubels, S.; Maes, A.; Baert, K.; Backer, P. D. Quantitative Determination of Salicylic Acid and Metabolites in Animal Tissues by Liquid Chromatography–Tandem Mass Spectrometry. *Anal. Chim. Acta* **2005**, *529* (1), 179-187.

4. Sánchez-Bonet, D. *et al.* RP-HPLC-DAD Determination of the Differences in the Polyphenol Content of *Fucus vesiculosus* Extracts with Similar Antioxidant Activity. *J. Chromatogr. B* **2021**, *1184* (122978).

5. Ceslova, L.; Pravcova, K.; Juricova, M.; Fischer, J. Rapid HPLC/MS/MS Analysis of Phenolic Content and Profile for Mead Quality Assessment. *Food Control* **2022**, *134* (108737).

6. Simon, R.; Priefer, U.; Pühler, A. A Broad Host Range Mobilization System for *in vivo* Genetic Engineering: Transposon Mutagenesis in Gram Negative Bacteria. *Nat. Biotechnol.* **1983**, *1*, 784-791.
